# Recognition of facial emotion expressions and perceptual processes in 22q11.2 deletion syndrome

**DOI:** 10.1111/eip.13295

**Published:** 2022-03-28

**Authors:** Antonino Buzzanca, Tommaso Accinni, Marianna Frascarelli, Eloisa Troisi, Georgios D. Kotzalidis, Carlo Di Bonaventura, Martina Fanella, Carolina Putotto, Bruno Marino, Massimo Pasquini, Massimo Biondi, Fabio Di Fabio

**Affiliations:** ^1^ Department of Human Neurosciences, Faculty of Medicine and Dentistry Sapienza University of Rome Rome Italy; ^2^ Faculty of Medicine and Psychology Sapienza University of Rome, Sant'Andrea Hospital Rome Italy; ^3^ Department of Paediatrics, Obstetrics and Gynaecology Sapienza University of Rome Rome Italy

**Keywords:** 22q11.2 deletion syndrome, facial emotional expression, positive and negative syndrome scale, schizophrenia, social cognition, social inference, visual‐spatial abilities

## Abstract

**Background:**

Social cognition (SC) deficits and of its facial emotion expression (FEE) component have been described in 22q11.2 Deletion Syndrome (22q11.2DS), a high‐risk for schizophrenia (SCZ) systemic genetic syndrome. Correlations between deficits in FEE skills and visual‐spatial abilities in people with 22q11.2DS warrant investigation.

**Methods:**

The sample consisted of 37 patients with 22q11.2DS (DEL), 19 with 22q11.2DS and psychosis (DEL‐SCZ), 23 with idiopathic SCZ, and 48 healthy controls. We assessed FEE through *The Ekman 60 Faces* test (EK‐F60), visual‐spatial skills with *Raven's Standard Progressive Matrices*, and symptom severity with the *positive And negative syndrome scale*. Statistics were conducted through multivariate analysis of variance and correlation analysis.

**Results:**

Patients with 22q11.2DS performed worse that the other groups in recognizing *Surprise*, *Disgust*, *Rage*, *Fear*, and Neutral expressions on the EK‐F60. Recognition of *Surprise* and *Disgust* correlated positively with visual‐spatial abilities in patients with 22q11.2DS; negative and cognitive symptoms correlated negatively with recognition of *Sadness*, *Surprise*, and *Disgust*.

**Conclusions:**

Patients with 22q11.2DS show impairments of both peripheral and central steps of the emotional recognition process, leading to SC deficits. The latter are present regardless of the presence of a full‐blown psychosis.

## 1 BACKGROUND

The 22q11.2 Deletion Syndrome (22q11.2DS) is associated to a rare autosomal dominant microdeletion at the 11.2 strand on the long arm (q) of chromosome 22; its incidence ranges from 1:3000 to 1:6000 births (McDonald‐McGinn et al., 2015). 22q11.2DS involves multiple organs and biological systems, characteristically showing cognitive and learning deficits (McDonald‐McGinn et al., 2015) (⅓ of patients show mild intellectual disability [Chow et al., 2006; Vorstman et al., 2015]), neurodevelopmental disorders like attention deficit/hyperactivity and autism spectrum disorders, anxiety disorders, and clinically important intellectual disabilities (Fiksinski et al., 2017). Lifetime incidence of psychosis in 22q11.2DS is 20 times greater than in the general population, reaching an overall prevalence of ≈25% (Bassett et al., 2003; Schneider et al., 2016). Mean onset age, symptoms, and treatment response do not significantly differ between idiopathic psychoses (e.g., schizophrenia [SCZ]) and psychosis in 22q11.2DS (Bassett et al., 2003). Due to the similarity of the neuropsychiatric features of 22q11.2DS and other psychoses, 22q11.2DS was assumed to represent a reliable biological model to study vulnerability factors of psychotic onset (Lattanzi et al., 2018).

Social cognition (SC) is a cognitive/emotional process‐based set of cognitive abilities aimed at representing and inferring others' mental state and intentions (Happé et al., 2017). A NIMH consensus workshop (Green et al., 2008) has defined the construct as built on five domains, that is, theory of mind (ToM), social perception, social knowledge, attributional bias, and emotional processing. This set of neurocognitive elements enable correct interpretation of social and relational contexts, and social rule and role inference (Frith, 2014). SC impairments were proposed as endophenotypical underpinnings of SCZ (Happé et al., 2017). The Italian Network for Research on Psychoses (Rocca et al., 2016) identified three patient clusters, based on their SC performance impairment severity, with the most impaired cluster involving the worst neurocognition, disorganization and positive symptoms. Network analysis showed SC deficits to be closer to core SCZ than positive, negative, and disorganization symptoms (Galderisi et al., 2020). Other than social inference deficits, neurocognitive impairments (Moss et al., 1999), motor delays (Swillen et al., 1999), emotion recognition and perspective taking (Badoud et al., 2017), and deficits in several neurocognitive abilities like attention (Sobin et al., 2005), executive functions (Campbell et al., 2010), working memory (Shapiro et al., 2014), visual‐spatial abilities (Simon et al., 2005), and social skills (Campbell et al., 2011; Ho et al., 2012) have been described in 22q11.2DS. Weinberger et al. (2016) found poor performances on all cognitive domains, comprising global neurocognitive and executive functions, episodic memory and SC in 22q11.2DS individuals, with the psychotic group showing the worst impairments. Jalbrzikowski et al. (2012) found negative correlations between ToM abilities and positive symptoms and between processing speed and negative symptoms in 22q11.2DS. Although a link with cognitive and executive deficits has been suggested, SC impairments were partially independent from global cognitive functioning (Campbell et al., 2015; Jalal et al., 2021). Neurocognition and SC were tightly associated in 22q11.2DS (Chow et al., 2006; Schneider et al., 2017; Yi et al., 2016).

Facial emotion expression (FEE) recognition is considered to be fundamental for SC, aiming at developing effective social inference abilities (McCabe et al., 2016).

Both SC and FEE impairments were described in 22q11.1DS; worse face recognition abilities were associated to more negative and paranoid symptoms (Schneider et al., 2017).

People with 22q11.2DS showed poorer ability in discriminating FEE intensity and their categorisation, compared with healthy controls (HCs; Leleu et al., 2016). We previously showed deficit in social inference in patients with 22q11.2DS, with or without psychosis, to be worse than in patients with SCZ, their unaffected siblings, and HCs (Frascarelli et al., 2020).

### 1.1 Aim of the study

Given these considerations, this study aimed at evaluating FEE recognition abilities in individuals with 22q11.2DS compared with a group of HCs and patients with idiopathic SCZ. Second, we sought to investigate potential correlations between FEE recognition ability and visual‐spatial processing abilities in the recruited groups. Finally, we set to explore potential correlations between FEE recognition and positive and negative symptom severity in the clinical samples. We chose to include two 22q11.2DS samples, with and without psychosis, to compare to patients with SCZ and HCs, because cognitive deficits exist in 22q11.2DS independently from psychopathology (Gao et al., 2018).

## 2 METHODS

The sample consisted of 42 individuals with 22q11.2DS without psychosis (DEL, N = 42), 24 individuals with 22q11.2DS with a diagnosis of psychosis (DEL‐SCZ, N = 24), 23 individuals with idiopathic SCZ (N = 23) and 48 HCs (N = 48). Thirteen patients of DEL‐SCZ group received a diagnosis of SCZ, three of them of Schizophreniform Disorder and eight of Psychotic Disorder Not Otherwise Specified; all participants of SCZ group received a diagnosis of SCZ. Participants' age was 17–50 years; they were consecutively enrolled at the specialized Outpatient Clinic for 22q11.2DS and at the Outpatient Clinic for Psychosis of the Department of Human Neurosciences at Policlinico Umberto I University Hospital, Sapienza University of Rome, from January 2017 to January 2018. HCs have been recruited by word of mouth. All participants signed free, informed consent before enrolment. The study received approval from the Ethics Committee of the Umberto I University Hospital, Rome, Italy. All data were anonymised. Data regarding type of antipsychotic treatment assumed by patients (i.e. missing, First Generation Antipsychotics, Second Generation Antipsychotics, or both) were collected for individuals with psychosis. Patient eligibility and psychotic disorder diagnosis (potentially including the diagnosis of SCZ, schizoaffective disorder, schizotypal personality disorder, delusional disorder, and brief psychotic disorder) were based on the Structured Clinical Interview for DSM‐IV Axis I Disorders/Patient Edition (First et al., 2016), with the aim to assess previous or current psychiatric symptoms. Genetic diagnosis was ascertained through Fluorescent In Situ Hybridization.

### 2.1 Assessment

Socio‐demographic variables, including age, job, social‐economic status, marital status, and education were collected during clinical interview. Patient symptoms were assessed by specifically trained clinicians using the following tools: *positive and negative syndrome scale* (PANSS) (Kay et al., 1987) to assess symptom severity; *Ekman's 60 Faces test* (EK‐60F) (Ekman & Friesen, 1975), consisting in showing each participant a set of pictures of actors (six women and four men) statically representing basic facial emotional expressions, that is, *rage (R)*, *fear (F)*, *sadness (SA)*, *happiness (H)*, *surprise (SU)*, *disgust (D)*, or *neutral (N)*. Each participant was then asked to state the recognized emotion. EK‐60F consists of two sections of 55 images each, with a maximum total score of 55; in this study, only section 1 was employed; Raven's *Standard Progressive Matrices* (SPM) to evaluate non‐verbal intelligence and the General Factor of fluid intelligence. This task consists of five sections (A to E), each involving 12 images to be completed with a lacking figure. Each participant is asked to choose the correct figure among different options, with increasing difficulty, based on a non‐verbal logic. Maximum total score is 60. We employed the first two sections of the SPM to assess the effectiveness of the visual‐perceptual components' organization. These sections reliably reproduce the complexity of FEE recognition (Waschl et al., 2017), which concerns the ability to elaborate visual‐spatial inputs rather than their simple recognition. The A and B SPM sections particularly evaluate the visualization process, intended as the ability to recognize visual patterns mentally reproducing their structure once transformed.

### 2.2 Statistical analysis

We employed Student's *t*‐test and Pearson's *Chi*‐square (*χ* ^2^) test for descriptive statistics. We analysed between‐groups differences in continuous demographical and clinical variables through four analysis of variance (ANOVA), assuming four‐levels group as the independent variable and respectively age, total IQ score, positive and negative symptom scores as the dependent variable in each analysis. Between‐groups differences in EK‐60F scores were analysed through multivariate ANOVA (MANOVA) with four‐level group as the independent variable and the seven EK‐60F scales' emotional recognition scores as the dependent variable. Effect analysis was conducted through Wilks' *Lamda*, Pillai's trace, Hotelling's trace, and Roy's root statistics. Post hoc results were corrected with Bonferroni correction for multiple comparisons. Correlation analyses were employed between performances in FEE recognition and visual‐spatial abilities and subsequently between performance on FEE recognition and positive and negative symptom severity. IQ and sex were included as covariates. Bonferroni‐corrected *p* < .05 was the cut‐off for statistical significance. We used the SPSS 25.0 version (Statistical Package for the Social Sciences, IBM Co., Armonk, New York, 2017) for all analyses.

## 3 RESULTS

DEL and DEL‐SCZ groups contained more males than the other two groups (*χ* ^2^ = 10.682; *p* = .015; Table 1). Groups differed for age (*F*_1,126_ = 7.467; *p* < .001) and post hoc analysis revealed that SCZ were older than the other three groups (*p* < .001), which did not differ in age. Differences in IQ total score between groups were significant (*F*_1,126_ = 36.880; *p* < .001) with HCs showing higher scores compared with the other groups (*p* < .001). No significant differences resulted between SCZ and DEL‐SCZ for exposure to different antipsychotic treatments (Table 1).

**TABLE 1:** Sociodemographic data of the recruited sample

	DEL‐SCZ N = 24		DEL N = 42		SCZ N = 23		HCs N = 48		Statistical test	
	N (%)		N (%)		N (%)		N (%)		*χ* ^2^	*p*
**Sex**										
Male	18 (75)		29 (69)		12 (52.2)		20 (41.7)		10.682	.015*
Female	6 (25)		13 (31)		11 (47.8)		28 (58.3)			
**Treatment**										
Missing	0 (0)				2 (8.7)				7.220	.065
SGA	19 (79.2)				21 (91.3)					
FGA	3 (12.5)				0 (0)					
FGA + SGA	2 (8.3)				0 (0)					

	Mean	SD	Mean	SD	Mean	SD	Mean	SD	*F*	*p*
**Age**	27.29	7.8	24.9	7.9	34.3	7.1	27.5	8.4	7.467	<.001**
**PANSS**										
Positive symptoms	19.7	5.6	9.5	1.8	14.2	4.4			54.625	<.001**
Negative symptoms	21.7	5.2	11.9	73.4	21	6.4			43.387	<.001**
General symptoms	44.6	8	28.8	5.1	36.9	8.2			40.267	<.001**
Total score	85.9	15.1	50.2	8.5	72.1	15.6			65.492	<.001**
**Total IQ**	79.0	17.9	86.9	18.1	87.6	19.8	116.8	22.5	36.880	<.001**

Abbreviations: DEL, individuals with 22q11.2DS; DEL‐SCZ, individuals with 22q11.2DS and psychosis; FGA, first generation antipsychotics; HCs, healthy controls; IQ, intelligence quotient; PANSS, positive and negative syndrome scale; SCZ, patients with idiopathic schizophrenia; SD, standard deviation; SGA, second generation antipsychotics; *statistical significant without Bonferroni‐correction; **statistical significant

after Bonferroni‐correction.

The groups differed on all PANSS score dimensions (Table 1). The DEL‐SCZ group scored higher in positive, negative, and general psychopathology scales (*p* < .001), and in total PANSS score (vs. SCZ, *p* = .001) than the other groups. DEL‐SCZ and SCZ groups did not differ in negative symptoms scale. SCZ group had higher scores than DEL group (*p* < .001) in all PANSS scales.

### 3.1 EK‐60F

**FIGURE 1:**
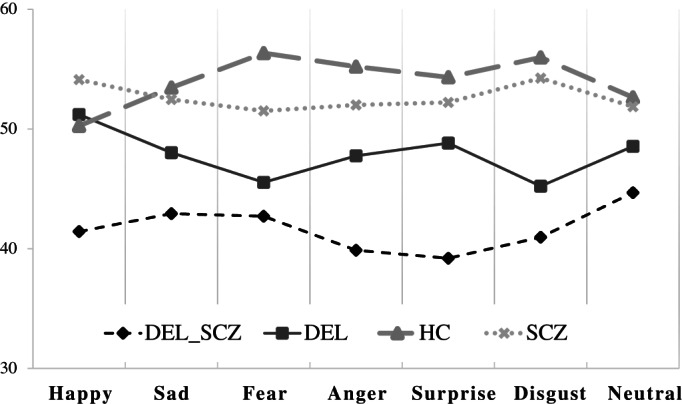
Standardized scores on the EK‐60F for patients with idiopathic schizophrenia (SCZ), participants with 22q11.2DS (DEL), participants with 22q11.2DS and psychosis (DEL_SCZ) and healthy controls (HC)

Mean T‐scores on the EK‐60F are shown in Table 2. MANOVA revealed significant between‐groups differences in EK‐60F tests (Wilks' *Lambda* = 0.409; *p* < .001). At univariate tests for subjects' effects, the *disgust* (Beta = 0.381), *rage* (β = 0.275), *fear* (β = 0.270), *surprise* (β = 0.255), and *neutral* (*p* < .05) variables were significant (*p* < .001). Post hoc analysis concerning the *happiness* variable showed significant differences between groups, with the DEL‐SCZ group performing worse than the DEL (*p* = .007), SCZ (*p* < .001), and HCs groups (*p* = .005) (Figure 1). Regarding the *sadness* variable, post hoc analysis revealed significant differences between the groups, with the DEL‐SCZ group performing worse than the SCZ (*p* = .01) and HC groups (*p* < .001); the DEL group scored significantly lower than HCs (*p* < .05). Regarding the *fear* variable, post hoc analysis revealed significant differences between groups, with the DEL‐SCZ group performing worse than the SCZ (*p* = .006) and HC groups (*p* < .001); for the *rage* variable, the DEL‐SCZ group performed worse than all other groups (*p* < .05) and the DEL group performed worse than the SCZ and HC groups (*p* < .05) (Figure 1); no significant differences were found between the SCZ and HCs groups on this variable. Similar results were found regarding the post hoc analyses for the *surprise* variable (*p* < .001). Post hoc analysis for the *disgust* variable showed the DEL‐SCZ group to perform worse than the SCZ and HC groups (*p* < .001); similar results were found for the DEL group (*p* < .001); no significant differences were found between the DEL‐SCZ and DEL groups. Post hoc analysis showed the DEL‐SCZ group to perform worse than HCs on the *neutral* variable (*p* = .023) (Figure 1).

**TABLE 2:** Mean T‐scores on the EK‐60F in the four groups

		HAPPY_T	SAD_T	FEAR_T	ANGER_T	SURPRISE_T	DISGUST_T	NEUTRAL_T
DEL‐SCZ	Mean	41.43	42.93	42.71	39.87	39.2	40.96	44.68
	SD	13.387	7.521	7.633	9.379	14.222	12.767	13.121
DEL	Mean	51.21	48.02	45.54	47.76	48.82	45.22	48.55
	SD	8.317	9.584	9.464	8.788	10.333	8.849	9.408
HCs	Mean	50.26	53.46	56.34	55.21	54.32	55.98	52.64
	SD	9.596	8.461	7.286	7.465	3.892	5.346	8.055
SCZ	Mean	54.12	52.47	51.51	52.01	52.22	54.25	51.86
	SD	6.553	11.931	9.602	10.057	6.925	6.651	10.245

Abbreviations: DEL, individuals with 22q11.2DS; DEL‐SCZ, individuals with 22q11.2DS and psychosis; HCs, healthy controls; SCZ, patients with idiopathic schizophrenia; SD, standard deviation.

### 3.2 Correlations between EK‐60F and SPM

Correlations were conducted between the seven basic emotions of EK‐60F and the total scores of the SPM‐A and SPM‐B series as variables. IQ and sex were considered as co‐variates. For the DEL‐SCZ group, correlations between SPM‐A and *rage* (*r* = 0.638; *p* = .004), *surprise* (*r* = 0.803; *p* < .001), *disgust* (*r* = 0.591; *p* = .01), and *neutral* (*r* = 0.540; *p* = .21) and between SPM‐B and *fear* (*r* = 0.594; *p* = .009), *rage* (*r* = 0.586; *p* = .011), and *surprise* (*r* = 0.480; *p* = .480) were significant. Significant were correlations in the DEL group between SPM‐A and *fear* (*r* = 0.460; *p* = .004), *surprise* (*r* = 0.414; *p* = .011), *disgust* (*r* = 0.421; *p* = .009), and *neutral* (*r* = 0.490; *p* = .490) and between SPM‐B and *fear* (*r* = 0.536; *p* = .001), *surprise* (*r* = 0.527; *p* = .001), *disgust* (*r* = 0.587; *p* = .587), and *neutral* (*r* = 0.673; *p* < .001) and in HCs, between SPM‐B and *fear* (*r* = 0.656; *p* = .022), *happiness* (*r* = 0.533; *p* = .016), and *neutral* (*r* = 0.671; *p* = .001).

### 3.3 Correlations between EK‐60F and PANSS

Correlations were conducted between the seven basic emotions of the EK‐60F and, respectively, positive symptom (seven items) and negative symptom (seven items) PANSS scores. Significant were correlations for the DEL‐SCZ group between *sadness* and item N1 (*r* = −0.673; *p* = .002), N2 (*r* = −0,742; *p* < .001), N3 (*r* = −0.763; *p* < .001), N4 (*r* = −0.663; *p* = .003), and between *rage* and item N3 (*r* = 0.627; *p* = .005), and between *happiness* and P2 (*r* = −0.486; *p* = .041), *surprise* and P2 (*r* = −0.497; *p* = .036), and *neutral* and P6 (*r* = 0.582; *p* = .011), for the DEL group, between *neutral* and N7 (*r* = −0.393; *p* = .022), *sadness* and P1 (*r* = 0.439; *p* = .009), and P3 (*r* = 0.374; *p* = .029), *fear* and P1 (*r* = 0.435; *p* = .010), and P3 (*r* = −0.416; *p* = .014), *disgust* and P2 (*r* = −0.509; *p* = .02), and for the SCZ group between *neutral* and N5 (*r* = −0.228; *p* = .046), and item P2 (*r* = −0.450; *p* = .031).

## 4 DISCUSSION

We aimed at investigating FEE recognition in patients with 22q11.2DS with and without psychosis, compared with individuals with idiopathic SCZ and HCs and found that individuals with 22q11.2DS showed impaired abilities in FEE recognition regardless of the presence of a full‐blown psychotic disorder. Evidence is accumulating that FEE recognition is deeply impaired in 22q11.2DS, in line with previous studies (Leleu et al., 2016; Peyroux et al., 2020; Zaharia et al., 2018).

Both SC and FEE recognition were shown to be impaired in psychosis, being associated to functioning impairments in SCZ (Barkl et al., 2014; Couture et al., 2006; Fett et al., 2011; Irani et al., 2012; Kohler et al., 2010). Our findings showed individuals with both 22q11.2DS and psychosis to display higher impairments in recognizing FEEs compared both to patients with SCZ and to HCs. Both DEL‐SCZ and SCZ groups were similarly impaired in neutral expression recognition compared with HCs, confirming reports that psychosis involves the inference of abnormal meanings from neutral expressions (Gao et al., 2021; Mitrovic et al., 2020; Silver et al., 2009).

According to Bruce and Young's (1986) model, EK‐60F focuses on peripheral components of emotion recognition, involving visual‐perceptual abilities interacting with central emotional processes to recognize FEEs. In line with previous studies (Peyroux et al., 2019), we observed specific impairments in recognition of *surprise* and *disgust* in the DEL group, compared with SCZ and to HCs; the DEL group also performed worse than HCs in recognition of *fear*, *rage*, *surprise*, and *disgust* expressions. Both DEL‐SCZ and SCZ groups showed significant impairment in the recognition of *fear* expressions compared with HCs. Patients with anxiety disorders tend to overestimate *fear* expression (Peschard & Philippot, 2017); we expected such result for people with 22q11.2DS, given their high comorbidity with anxiety disorders (Fabbro et al., 2012; McDonald‐McGinn et al., 2015). We presume that impaired visual‐spatial abilities in 22q11.2DS countered the overestimation of fear.

Overall, DEL‐SCZ and DEL groups did not show differences in recognition of *sadness*, *fear*, *disgust*, and *rage* expressions, although impairments in emotion recognition have been reported during early stages of psychotic disorders (Barkl et al., 2014). It appears that full‐blown psychosis does not significantly worsen FEE recognition deficits, which in turn appear to be tightly associated to the syndrome and may be likely considered as endophenotypes of psychosis, as already suggested (Comparelli et al., 2013).

Consistently with literature (Schneider et al., 2017; Vangkilde et al., 2016), we found positive correlations between SC deficits and PANSS measures. SPM‐A and SPM‐B series were employed to investigate emotion recognition in relation to its central decoding step; indeed, FEE recognition involves similar patterns of the same basic structure required to effectively decode visual information, in addition to correct perceptual processes. The A series focuses on immediate visual perception, while the B series on the capacity to elaborate acquired information (Waschl et al., 2017). For the DEL and the DEL‐SCZ groups, the recognition of *Surprise* and *Disgust* positively correlated with the SPM‐A series, while the *Fear* recognition correlated with the SPM‐B series, suggesting high involvement of general visual‐spatial abilities in emotion recognition, regardless of the presence of psychosis (Peyroux et al., 2019; Peyroux et al., 2020). 22q11.2DS patients' deficits in elaborating visual‐spatial information likely impair the central stage of the emotional recognition process, as implemented by the amygdala (Baxter & Croxson, 2012), partially explaining the unexpected lack of *Fear* overestimation. Globally, impairments in both peripheral perceptual and visual‐spatial functions are likely to interact with each other in 22q11.2DS, eventually resulting in the observed emotion recognition deficits.

Regarding *Sadness* and *Surprise* recognition, the DEL‐SCZ group showed low emotional and affective resonance, as highlighted by PANSS symptoms N1 (*blunted affect*), N2 (*emotional withdrawal*), N3 (*poor rapport*), N4 (*passive/apathetic social withdrawal)*, N5 (*Difficulty in abstract thinking*), and P2 (*Conceptual disorganization*), in turn leading to deficits in central emotional decoding, in line with previous psychosis literature (Comparelli et al., 2013; Edwards et al., 2001; Leung et al., 2011; Won et al., 2019). In the DEL group, likely due to lower negative symptom severity, the correlation between error rates in *Sadness* recognition and low emotional resonance was not observed. In the same group, *Disgust* recognition negatively correlated with disorganization (P2, *conceptual disorganization* and N7, *stereotyped thinking*), as already reported (Kohler et al., 2000). Considering that emotion recognition deficits and psychotic symptom severity did not correlate in the SCZ group, we may hypothesise a 22q11.2DS endophenotype underlying emotional decoding deficits, regardless of psychotic symptoms. This endophenotype would likely interact with psychotic symptoms once they emerge.

The main limitation of the study is the low sample size, but 22q11.2DS is a rare genetic syndrome with low incidence, which is difficult to recruit, addressed with a strong statistical analysis. The correlations we here employed described associations between neurocognitive processing and symptomatology in 22q11.2DS but are unable to allow us to infer causal relationships between the considered variables, due to the cross‐sectional design of the study. Longitudinal studies could better address whether FEE recognition defines a risk factor for developing psychosis.

DEL patients appeared high‐functioning likely due to the level of assistance they are exposed to (regular follow‐ups, pharmacological treatments, cognitive remediation programs), whereas patients with low‐level functioning may not have been recruited given their inability to undergo the study tasks.

## 5 CONCLUSIONS

FEE recognition appears to be impaired in 22q11.2DS, regardless of concurrent psychosis. Recognition of *surprise*, *fear*, and *disgust* emotions positively correlated with visual‐spatial abilities, suggesting a direct involvement in emotion recognition of central decoding processes. This study confirmed tight relations between emotion recognition abilities as part of SC and neurocognitive processes, such as visual‐spatial ones. Focusing on SC deficits in syndromes representing suitable models to study psychosis (Lattanzi et al., 2018) could be useful in enforcing preventive and therapeutic programmes apt to correct such deficits (Pine et al., 2021; Shashi et al., 2012; Shashi et al., 2015).

## CONFLICT OF INTEREST

The authors declare no conflict of interest.

## Data Availability

The data that support the findings of this study are available on request from the corresponding author. The data are not publicly available due to privacy or ethical restrictions.
